# Processing speed and working memory are predicted by components of successful aging: a HUNT study

**DOI:** 10.1186/s40359-022-00718-7

**Published:** 2022-01-28

**Authors:** Ingunn Bosnes, Ole Bosnes, Eystein Stordal, Hans M. Nordahl, Tor Å. Myklebust, Ove Almkvist

**Affiliations:** 1 Clinic for Mental Health and Substance Abuse, Namsos Hospital, Nord-Trøndelag Hospital Trust, 7800 Namsos, Norway; 2 Department of Psychology, Norwegian University of Science and Technology (NTNU), Trondheim, Norway; 3 Department of Mental Health, Norwegian University of Science and Technology (NTNU), Trondheim, Norway; 4 Division of Mental Health, St. Olavs Hospital, Trondheim University Hospital, Trondheim, Norway; 5 Department of Registration, Cancer Registry Norway, Oslo, Norway; 6 Department of Research and Innovation, Møre and Romsdal Hospital Trust, Ålesund, Norway; 7 Department of Psychology, Stockholm University, Stockholm, Sweden; 8 Department of Neurobiology, Care Sciences and Society, Karolinska Institutet, Stockholm, Sweden

**Keywords:** Aging, Successful aging, Cognition, Health

## Abstract

**Background:**

Research has demonstrated that cognitive heterogeneity occurs with aging both within and between individuals. The purpose of this study was to explore whether the cognitive heterogeneity in aging was related to the subgroups of successful and usual aging.

**Method:**

Participants were a representative sample of normal older adults (n = 65, age range 70–89 years). All subjects had participated in the third phase of the Nord-Trøndelag Health Survey (HUNT3) and completed all subtests in the Wechsler Memory Scale (WMS-III) and Wechsler Adult Intelligence Scale (WAIS-III). Successful aging was defined in four ways in the study: as (1) absence of disease, (2) high functioning, (3) active engagement with life, or (4) all three components combined. Five domains of memory and intelligence functions were investigated using linear regression analysis, with group membership (successful versus usual aging) as predictors and age, sex and education as correlates.

**Results:**

Processing speed performance was correlated with the successful aging component absence of disease, younger age and being of the female sex, while working memory performance was correlated with the successful aging component absence of disease and more years of education. Performance in other domains (verbal, visuospatial, and episodic memory) were not related to any successful aging definition. Age had a consistent negative effect on the processing speed domain for all successful aging definitions. Education was positively linked to cognitive performance on the verbal and working memory domains. Being female was positively linked to processing speed and episodic memory.

**Conclusions:**

Processing speed and working memory were linked to successful aging when it was defined as absence of disease, but not by other components of successful aging, i.e. domain-specific. In contrast, other cognitive domains were not related to any components of successful aging.

**Supplementary Information:**

The online version contains supplementary material available at 10.1186/s40359-022-00718-7.

## Background

With world-wide population aging, it is important to identify the specific aspects of ‘successful’ aging (SA). Research has suggested that some cognitive decline can be expected with advancing age, particularly in the late sixties and early seventies, and that this decline will vary across the cognitive domains and between individuals [1–4]. So-called ‘crystallized’ abilities such as verbal ability often remain stable until late in life [3, 5, 6], while ‘fluid’ functions such as processing speed, episodic memory, working memory and executive function decline with advancing age [3, 6, 7].

Heterogeneity within aging, manifested as increasing diversity of health and functioning among individuals, can be quantitatively described as, or related to, (1) health or the presence of manifest or covert disease, (2) differing degrees of frailty [8], (3) the presence or absence of life satisfaction, or diverse other factors [9]. The heterogeneity of aging can also be described using categorical models like the Rowe and Kahn model of SA, in which normally aging older adults are classified as undergoing ‘usual’ aging (UA) or SA [10]. SA has been defined as fulfilling the criteria for three components: a low probability of disease and disability (component I), high cognitive and physical functioning (component II), and active engagement with life (component III) [11, 12]. Those who are undergoing UA have some decline in health and increased risk of disease and disability, often seen as typical age-associated functional decline [13, 14]. In our opinion, the SA model has been important for the growing interest and research on factors related to normal aging. However, the model can be criticized for its weight on diseases and too little focus on layman perspectives [15] and for not fully appreciating the importance of structural factors for health like income and other factors [16].

A previous study based on the large population-based Nord-Trøndelag Health Survey (HUNT) showed that the three SA components in the Rowe and Kahn model had only weak correlation with each other [17]. Similar findings have been reported in other studies [18, 19]. The components can be considered as different aspects of aging or summarized together as an index of frailty. The relatively low correlation between the components means it is important to investigate correlates of both each separate component and the unified SA construct (all three components fulfilled).

In the MacArthur studies of SA, high physical and cognitive functioning was often defined as achieving results in the top one-third in screening tests [20, 21]. However, to our knowledge, Rowe and Kahn have not precisely characterized the definition of high cognitive functioning across different cognitive domains with respect to SA and UA. The difference in cognition between SA and UA subgroups could be general and invariant across combined cognitive domains, or it could be specific and variable by domain.

The hypothesized differentiation of normal aging into SA or UA according to a general effect across cognitive domains is supported by previous research showing that normal adults can be classified into subgroups that differ in mean performance across domains [22, 23]. The alternative concept of domain-specific differences is in agreement with the variable cognitive decline seen in different domains [3, 6, 7, 24]. Departing from Cattell’s (1963) concepts of fluid and crystallized functioning [25], it could be hypothesized that individuals classified as SA or UA differ in a relatively invariant manner across the cognitive domains, i.e., the classification is group-related. Or, alternatively, it could be that SA and UA individuals differ not only by group, but also according to variations across domains in a domain-by-group interaction, i.e., the classification is domain-related.

This study investigates the associations between cognitive domains, measured by the WMS-III/WAIS-III [26], and a SA model inspired by the Rowe and Kahn model of SA/UA subgroups in normal aging. It is hoped that the study of potential correlations between performance on these well-established tests and the three components of a modified Rowe and Kahn model will help to clarify their possible empirical association with cognition. The study is explorative and therefore no specific hypotheses are set forth, although we generally expect that age has a negative and education has a positive association with cognition. The study investigates whether performance on the WAIS-III and the WMS-III is correlated with classification of individuals into the SA and UA subgroups when these are defined based on (a) all three components together or (b) each of the three components separately. To our knowledge, studies relating SA/UA differentiation to a comprehensive assessment of cognition have not been performed previously. Thus, the study will improve our understanding of the role of cognition in SA.

## Methods

### Participants

A sample of 65 community-dwelling older adults who had participated in the HUNT3, as well as in a study of intelligence and memory about 2 years later, was recruited to the present study. HUNT is a large population-based epidemiological health survey of all (about 125 000) inhabitants in the county of Nord-Trøndelag in Norway. There have been four phases of the HUNT over the period from 1984 to 2018. The surveys have had a high or acceptable participation rate and data drawn from the samples are therefore able to be extrapolated to the Norwegian population [27]. All study participants had participated in the HUNT3 (2006–08) as well as in a study of intelligence and memory about two years later [28]. Inclusion criteria for our study were: (1) age 70–89 years, and (2) living near the examination site. Exclusion criteria for our study were similar to those used in the US Standardization studies for WAIS-III/WMS-III [26]. Exclusion criteria included: uncorrected hearing loss, uncorrected visual impairment, current treatment for alcohol or drug dependence, consumption of more than three alcoholic beverages on more than two nights a week, seeing a doctor or other professional for memory problems or problems with thinking, upper extremity disability, any period of unconsciousness for 5 min or more, head injury resulting in hospitalization for more than 24 h, reporting medical or psychiatric conditions that could potentially affect cognitive functioning (for example stroke, epilepsy, multiple sclerosis, Alzheimer’s dementia and more), or currently taking antidepressant, antianxiety, or antipsychotic medication.

Individuals who were 70 years or older, and had complete HUNT3 data on daily functioning, were recruited to the present study. The sample characteristics can be seen in Table 1. The sex and age distribution in the study population was comparable to that in the HUNT3 population overall (*p* > 0.1), but the study sample was significantly better educated (*χ*^2^(2) = 13.25, *p* < 0.001) than the HUNT3 population.

**Table 1:** Demographic characteristics of the study sample and the general HUNT3 population

	Study sample	HUNT3
N	65	7975
Age^a^ (Mean ± SD^b^), y	77.6 ± 5.0	77.0 ± 4.9
Age range, y	70.0–86.6	70.0–89.9
Females (%)	54	55
Education		
Elementary school, n (%)	21 (32)	4232 (53)
High school, n (%)	26 (40)	1902 (24)
College/university, n (%)	9 (14)	811 (10)
Missing, n (%)	9 (14)	1030 (13)

^a^Age on joining the HUNT3

^b^SD, standard deviation

### Procedure

Potential participants for the study of intelligence and memory were drawn from the HUNT3 database. They were telephoned consecutively, until at least 20 individuals in each of six age bands (55–64, 65–69, 70–74, 75–79, 80–84, and 85–89 years) gave consent to participate. Before testing, all potential participants underwent a structured clinical interview about current diseases that would make them unsuitable for participation in the study. The interviews revealed that one individual had recently been diagnosed with dementia and another had suffered a cerebral infarction. These two individuals were excluded. From this original sample of 122 individuals 65 individuals who were 70 years or older, and had complete HUNT3 data on daily functioning, were recruited to the present study.

### The definition of Successful Aging

Following the Rowe and Kahn conceptualization and previous research, SA was defined as a three-component construct: (I) absence of major disease, (II) high functioning, and (III) active engagement with life [12, 19, 29, 30]. The classification of participants into the SA or UA groups was based on self-reported health information from the HUNT3 Study. The different components were operationalized as follows:

Absence of major disease (component I) was defined as absence of a self-reported history or presence of any of the following diseases: myocardial infarction, angina pectoris, heart failure, other heart disease, stroke/brain hemorrhage, chronic bronchitis, emphysema or chronic obstructive pulmonary disease (COPD), diabetes, or cancer; and the absence of depression, defined as a score < 8 on the depression subscale of the Hospital Anxiety and Depression Scale (HADS-D) [31, 32]. A valid rating of HADS-D was defined as five or more completed items on the HADS-D. For respondents who had answered five or six items, the completed sum was multiplied by 7/5 or 7/6. This procedure has been used in previous HUNT-related studies [32, 33]. Absence of disease was coded 1 and presence of disease 0.

High functioning (component II) was defined as being able to perform the following activities independently: walk around indoors on the same floor, go to the toilet, wash themselves, take a bath or shower, dress and undress, go to bed and get up, eat, prepare warm meals, do light house-work (e.g.: wash dishes), do heavier house work (e.g.: wash floors), do the laundry, do the shopping, pay bills, take medicines, go out, and take the bus. Respondents reporting an inability to perform one or more of these activities independently were recorded as having impaired physical functioning. As cognition was studied as a separate factor, cognitive measures were not included in the definition of component II.

Engagement with life (component III) was described by Rowe and Kahn (1998) as being both actively related to other people and productive in society (i.e. doing paid or unpaid work). Respondents were classified as being actively engaged with life if they (1) were currently in paid or unpaid work or (2) had gone to a museum/art exhibition, a concert, the theater, a film, church/chapel, or a sporting event or had participated in community service, a choir, theater work or church work at least once a month over the last six months. All questions had to be answered negatively to be classified as non-active coded 0.

### Cognitive assessment

A total of 16 subtests from the WAIS-III/WMS-III related to five cognitive domains were selected for this study. Eight subtests of the WAIS-III (Picture Completion, Vocabulary, Digit Symbol-Coding, Similarities, Block Design, Matrix Reasoning, Information, and Symbol Search) and eight subtests of the WMS-III (Logical Memory, Verbal Paired Associates, Visual Reproduction, Faces, Family Pictures, Letter-Number Sequencing, Digit Span and Spatial Span) were used in the study. The subtests Vocabulary and Information are tests of crystallized abilities [34], Block Design and Matrix Reasoning are tests of fluid reasoning [26], while Digit Symbol-Coding and Symbol Search are tests of processing speed. The subtests Logical Memory, Verbal Paired Associates, Visual Reproduction, Faces and Family Pictures are considered as measures of fluid functioning, while Letter-Number Sequencing, Digit Span and Spatial Span can be seen as measures of working memory. All tests were administered and scored according to standardized guidelines [26]. As previous studies on the factor structure of the WMS-III have shown that the immediate and delayed memory tests are highly associated to the same factor [35, 36], only immediate memory subtests were used in this study.

There was a time interval between the HUNT Study and the cognitive testing. Because the time interval between the HUNT3 and the cognitive testing varied across individuals from 1.5 to 3.9 years, individually age-adjusted test results for each objective cognitive test were calculated, using linear regression of the original test result plus time interval x beta for age regression in the whole sample for each test. The age-adjusted scores were used in all analyses.

### Statistical analysis

Statistical analyses were performed with STATA version 15.0 software [37]. Descriptive statistics were used to present background information (Table 1). Because of the time interval between the HUNT3 and cognitive testing, all cognitive test results were individually age-adjusted to the time that the individual attended the HUNT3 examination. The relationship between each component and age was analysed for the SA and UA subgroups using the Pearson correlation. The cognitive test results were combined to the WAIS-III/WMS-III factor structure by means of one PCA including verbal, visuospatial and processing speed tests and a second PCA including episodic and working memory tests in a sample of normal aging adults 55–90 years of age recruited similarly to the present study (presented in a Additional file 1: Panel A and B). The verbal domain consists of the subtests Vocabulary, Similarities and Information, the visuospatial domain consists of the subtests Block Design, Matrix Reasoning and Picture Completion, the processing speed domain of the subtests Symbol Digit and Symbol Search, the episodic memory domain consists of the subtests Logical Memory, Visual reproduction, Faces and Family Pictures while the working memory domain consists of the subtests Letter-Number-Sequencing, Digit Span and Spatial Span. The cognitive domains were subjected to a linear regression analysis with group membership (SA or UA) as predictors and age, sex, and years of education as covariates of cognitive domains.

## Results

### SA classification

Classification of participants into the SA or UA groups was achieved in four ways, either based on all three SA components or on each of the three separate components. When all three components were included in the SA definition, there were 28 participants in the SA subgroup and 37 in the UA subgroup (43% vs 57%). When SA was defined by component I, absence of disease, there were 41 SA and 24 UA (63% vs 37%); component II, no impairment in daily functioning, there were 56 SA and 9 UA (86% vs 14%); and component III, engagement with life, there were 44 SA and 21 UA (67% vs 32%); see Table 2.

**Table 2:** Demographic characteristics for participants after defining group membership (SA vs UA) according to the unified construct based on all three components or separate components based on health, high daily functioning or engagement with life

Characteristic	SA	UA
**All three components**		
n	28	37
Age (Mean ± SD), years	78.8 ± 5.0	81.2 ± 5.5
Female/male, n	17/11	18/19
Education (Mean ± SD), years	9.7 ± 2.2	10.0 ± 2.8
**Absence of disease criteria**		
n	41	24
Age (Mean ± SD), years	81.5 ± 5.2	79.5 ± 5.4
Female/male, n	23/18	12/12
Education (Mean ± SD), years	9.9 ± 2.4	9.8 ± 2.8
**High functioning criteria**		
n	56	9
Age (Mean ± SD), years	79.8 ± 5.4	82.9 ± 4.9
Female/male, n	31/25	4/5
Education (Mean ± SD), years	10.0 ± 2.6	8.9 ± 2.2
**Engagement with life criteria**		
n	44	21
Age (Mean ± SD), years	79.9 ± 5.3	80.9 ± 5.7
Female/male, n	24/20	11/10
Education (Mean ± SD), years	9.9 ± 2.6	9.7 ± 2.5

SA, ‘successful’ aging; UA, ‘usual’ aging

There were no significant group effects for age (*p* > 0.1), sex distribution (*p* > 0.1) or years of education (*p* > 0.1) for any SA/UA definition.

### SA subgroups, age, sex and years of education and the cognitive domains

The outcome of linear regression analyses for each of the five cognitive domains with four predictors are presented in Table 3: (1) subgroups of SA and UA based on four definitions of subgroup membership, (2) age, (3) sex, and (4) years of education.

**Table 3:** Outcome of linear regression analysis on five cognitive domains as dependent variable and the SA constructs as predictors and age, sex and years of education as correlates

Domain	All three components combined Beta (95% CI), *p* value	Component I, Absence of disease Beta (95% CI), * p* value	Component II, High functioning Beta (95% CI), * p* value	Component III, Active engagement Beta (95% CI), * p* value
Verbal function				
SA	− 0.19 (− 0.58, 0.20)	− 0.36 (− 0.74, 0.03)	− 0.37 (0.93, 0.19)	0.13 (− 0.27, 0.53)
Age	0.00 (− 0.03, 0.04)	0.00 (− 0.03, 0.04)	0.00 (− 0.03, 0.04)	0.01 (− 0.03, 0.05)
Sex	0.19 (− 0.21, 0.59)	0.18 (− 0.21, 0.57)	0.17 (− 0.22, 0.57)	0.22 (− 0.17, 0.62)
Education	0.25 (0.17, 0.33)***	0.26 (0.18, 0.33)***	0.27 (0.18, 0.34)***	0.26 (0.18, 0.33)***
Visuospatial				
SA	− 0.06 (− 0.58, 0.47)	0.02 (− 0.51, 0.55)	0.04 (− 0.71, 0.79)	0.08 (− 0.46, 0.61)
Age	− 0.01 (− 0.07, 0.04)	− 0.01 (− 0.06, 0.04)	− 0.01 (− 0.06, 0.04)	− 0.01 (− 0.06, 0.04)
Sex	0.45 (− 0.09, 0.98)	0.46 (− 0.08, 0.99)	0.46 (− 0.08, 1.00)	0.46 (− 0.07, 0.99)
Education	− 0.05 (− 0.16, 0.05)	− 0.05 (− 0.16, 0.05)	− 0.05 (− 0.16, 0.05)	− 0.05 (− 0.16, 0.05)
Processing speed				
SA	0.35 (0.00, 0.70)	0.39 (0.05, 0.74)*	0.43 (− 0.07, 0.93)	0.11 (− 0.25, 0.47)
Age	− 0.05 (− 0.08, − 0.01)**	− 0.05 (− 0.08, − 0.02)**	− 0.05 (− 0.08, − 0.02)**	− 0.06 (− 0.09, − 0.02)**
Sex	− 0.67 (− 1.02, − 0.31)***	− 0.68 (− 1.03, − 0.34)***	− 0.67 (− 1.03, − 0.32)***	− 0.72 (− 1.08, − 0.36)***
Education	0.02 (− 0.05, 0.09)	0.02 (− 0.05, 0.09)	0.01 (− 0.06, 0.08)	0.02 (− 0.05, 0.09)
Episodic memory				
SA	0.04 (− 0.37, 0.46)	− 0.23 (− 0.64, 0.19)	0.17 (− 0.43, 0.76)	0.21 (− 0.21, 0.63)
Age	− 0.03 (− 0.07, 0.02)	− 0.03 (− 0.07, − 0.01)	− 0.02 (− 0.06, − 0.02)	− 0.02 (− 0.06, 0.01)
Sex	− 0.48 (− 0.90, − 0.05)*	− 0.51 (− 0.93, − 0.09)*	− 0.47 (− 0.89, − 0.04)*	− 0.47 (− 0.89, − 0.06)*
Education	0.03 (− 0.05, 0.12)	0.03 (− 0.05, 0.12)	0.03 (− 0.05, 0.11)	0.03 (− 0.05, 0.12)
Working memory				
SA	0.28 (− 0.11, 0.67)	0.40 (0.02, 0.79)*	0.22 (− 0.35, − 0.78)	0.15 (− 0.26, 0.55)
Age	− 0.00 (− 0.04, 0.04)	− 0.00 (− 0.04, 0.04)	− 0.01 (− 0.04, 0.03)	− 0.01 (− 0.05, 0.03)
Sex	0.24 (− 0.16, 0.64)	0.24 (− 0.16, 0.62)	0.22 (− 0.19, 0.62)	0.20 (− 0.20, 0.60)
Education	0.09 (0.01, 0.17)*	0.09 (0.01, 0.16)*	0.08 (− 0.00, 0.16)	0.08 (0.00, 0.16)*

Significant effects (*p* < 0.05), 95% CI and beta weights are presented in bold type. *p < 0.05; **p < 0.01; ***p < 0.001

When the subgroups were defined in terms of the absence of disease component, but not for any other definition, the difference between groups was significant (in favor of SA vs UA) for the processing speed and working memory tests.

Higher education had a significant positive effect on verbal function. Neither age, sex nor years of education had a significant effect on visuospatial function. Higher age had a significant negative effect on processing speed, whereas female sex had a positive effect on processing speed and episodic memory performance. Higher education had a positive effect on working memory performance, whereas neither age nor sex had a significant effect on the working memory tests. To summarize, years of education was a significant predictor of seven of 20 outcomes, age was a significant predictor of cognitive function in four of 20 outcomes, sex in eight of 20 outcomes, while subgroup was a significant predictor in two of 20 outcomes. The majority of the significant effects were observed in the processing speed, working memory and episodic memory domains, while significant effects were lacking in the visuospatial domain.

## Discussion

In this study, we explored the extent to which cognitive heterogeneity in normal aging was related to SA and UA (inspired by the Rowe and Kahn SA model). The most important results were that processing speed and working memory results were associated to the SA and UA subgroups when the latter were defined by the criterion related to absence of disease, whereas verbal, visuospatial, and episodic memory abilities were not significantly predicted by the SA and UA subgroups for any definition. This means that there was a selective cognitive domain-related group difference rather than a general group difference, which suggests that the SA components are differently associated with cognitive function. Further, our findings support the statement by Rowe and Kahn (1998) that “physical and mental abilities are substantially independent of each other”. The findings also support our opinion that each of the SA criteria should be studied both by itself and in combination with the others.

### Cognitive domains and SA/UA

When SA was defined as absence of disease, we found a significant difference in cognitive ability between the SA and UA subgroups when it comes to processing speed and working memory performance. The absence of disease component included cardiovascular diseases, diabetes, COPD, cancer and depression. Cardiovascular disease and depression are related to cognitive decline [4], and better cognitive performance could therefore be expected by being SA. Processing speed is well-known from previous research to be sensitive to cognitive aging [38] and has been proposed as a possible common underlying factor of cognitive aging [39, 40]. The relation to cognition may be either through a direct link or it may be mediated through underlying risk factors and lifestyles. Cardiovascular disease (included in the disease component) have been shown to share common risk factors with dementia, and even though processing speed is a function sensitive to aging, cardiovascular risk factors and cardiovascular disease might accelerate the cognitive decline [41, 42], while absence of cardiovascular disease or other chronic diseases have been related to less risk of cognitive decline [38].

Working memory involves short-term storage and manipulation of information involved in diverse cognitive activities. Our result is in line with theories of memory, where working memory is considered to be of crucial importance for learning and memory [43]. Possibly this mental capacity is also essential for carrying out activities in daily life, particularly activities that require mental operations like divided attention, planning and keeping track. This line of thinking is supported by a study of different measures of working memory in the WAIS-IV, which reported that measures requiring higher active control (like Letter-Number Sequencing) were most strongly related to general cognition in older adults [44]. Therefore, working memory seems to be an important factor for general cognition, i.e. intelligence and intellectual efficiency across aging [45]. Intelligence is known to be a powerful predictor of future health and mortality [46]. This knowledge supports the present finding that working memory performance was associated with the absence of disease component.

When SA was defined as high functioning, we found no significant group differences between the SA or UA subgroups in cognitive performance. Possibly the result is related to the exclusion criteria, as seriously impaired daily functioning would have resulted in exclusion from the study and as a result few individuals reported impaired functioning. Rowe and Kahn (1998) suggested that a combination of three criteria represented SA most fully. However, our findings suggest that the components are relatively independent of each other and they appear to measure different aspects of health in normal aging. This is in line with previous research showing a low correlation between the components [17–19].

When the definition of SA and UA was based on engagement with life, there were no significant differences between the subgroups with respect to cognitive functioning. Social engagement measured as frequency of social activity and social support has previously been linked to global cognition [47], which was a summary measure of subtests of episodic memory, semantic memory, working memory, perceptual speed and visuospatial ability. However, we found no relation between SA based on engagement in life and cognitive function in this study.

Finally, the Rowe and Kahn concept of SA is categorical and it has been suggested that the concept should be enlarged by including comprehensive subjective aspects of aging well [9, 48] in addition to the three suggested components. A recent study examining five different SA models found that a model containing a range of SA criteria (14 medical conditions; ADL ability; cognitive health; well-being; social engagement) fit the model better and had a better construct validity than a purely biomedical construct or a purely psychosocial construct. Both that study and ours (see below) made it clear that aging is a significant factor in addition to the three SA components.

### Sensitive cognitive domains

The most sensitive cognitive domains related to SA/UA were processing speed and working memory. This indicates a selective vulnerability of cognition in the SA and UA subgroups, which may be related to brain health and integrity [49]. The selection criteria for our study were intended to exclude individuals with conditions that could affect cognition. However, this does not rule out that some of the individuals classified as belonging in the UA subgroup in this study have had incipient cardiovascular disease [50, 51] and other categories of incipient disease [52].

In contrast to the significant difference between the SA and UA groups in the processing speed and working memory domains, there were no differences in the other domains for any of the four definitions of SA and UA. The finding that verbal function was preserved or even higher with advancing age, demonstrates that there are age-insensitive cognitive functions. That performance in verbal functions like vocabulary and general knowledge resist the effects of aging well is well known from previous research [3, 5, 6].

Visuospatial cognition seems to represents a separate category of cognitive function, as there were no group-based differences in visuospatial cognition or effects due to age, sex or education.

### General factors influencing cognitive aging

There was a positive effect of education on verbal function and working memory for most SA/UA definitions. There was also a positive effect of female sex on episodic memory and processing speed. There was, as expected, a negative effect of age on processing speed [53]. The effect of age did not depend on the definition of SA and UA, but could have been caused by other factors. In a recent study on biological aging, it was suggested that aging occurs on many levels including basic biological (for instance DNA changes), physiological, cognitive and functional processes, with varying trajectories across ages [54]. This suggests that basic biological processes should perhaps be added to the Rowe and Kahn components in order to more fully understand the differences between SA and UA.

### Strengths and limitations

The main strength of this study is that it is one of few studies that have investigated the effects between the subgroups of normal aging (SA and UA) and a comprehensive assessment of cognitive performance. Another strength is that five cognitive domains were assessed by using many tests from the WAIS-III and the WMS-III, which are well validated cognitive measures, while previous studies typically used screening tests or short test batteries. Thirdly, the sample was recruited from a large population-based survey of health and lifestyle. Fourthly, strict exclusion criteria were used and it is unlikely that individuals with pathological aging were included.

However, several limitations should also be noted. Firstly, the statistical power was sufficient, but was at the low end with regard to detecting significant effects. Secondly, a prospective cross-sectional design was used, giving a snapshot rather than a true picture of changes in aging; hence, no causal inferences can be drawn. Thirdly, individuals in our sample had to fulfill the inclusion criteria of the cognitive assessment study, which required adequate physical functioning and may have led to a higher proportion of SA in the sample than in the general population. Fourthly, there was a gap between classification into SA or UA groups and assessment of cognition and memory. However, to minimize the associated risk, the cognitive test results were age-adjusted to the time point of the HUNT3, prior to analysis.

## Conclusions

The main finding was that processing speed and working memory were significantly associated with the SA component absence of disease. This effect was domain-specific and related to fluid cognition. This may be a step forward in understanding the role of cognition in SA. However, age, sex and education were the predominant correlates of high cognitive function compared to the components.

## Supplementary Information

**Additional file 1.** **Panel A.** Rotated component matrix for eight WAIS-III cognitive tests (panel A). Loadings > 0.60 are in bold type. **Panel B.** Rotated component matrix for eight WMS-III memory tests (panel B). Loadings > 0.60 are in bold type.

## Data Availability

The public sharing of the data set used in this study has been restricted by the Regional Committees for Medical and Health Research Ethics (post@helseforskning.etikkom.no) in accordance with Norwegian law, as HUNT survey (http://www.hunt.no) participants have not given consent to the public sharing of their data. The data are therefore available upon appropriate request to the Data Access Committee at the HUNT Research Centre (email: hunt@medisin.ntnu.no).

## Abbreviations

HADS-D: The Hospital Anxiety and Depression Scale, depression subscale
HUNT3: The third phase of the Nord-Trøndelag Health Survey
SA: Successful aging
UA: Usual aging
WAIS-III: The Wechsler Adult Intelligence Scale, third edition
WMS-III: The Wechsler Memory Scale, third edition
