# Supplementary material for: Processing speed and working memory are predicted by components of successful aging: a HUNT study
Source: BMC Psychol. 2022 Jan 28;10:16. doi: 10.1186/s40359-022-00718-7 (PMC8800254; doi:10.1186/s40359-022-00718-7)
Supplement: Supplementary file 1 — Additional file 1. Panel A. Rotated component matrix for eight WAIS-III cognitive tests (panel A). Loadings > 0.60 are in bold type. Panel B. Rotated component matrix for eight WMS-III memory tests (panel B). Loadings > 0.60 are in bold type. [file 40359_2022_718_MOESM1_ESM.docx]

**Panel A.** Rotated component matrix for eight WAIS-III cognitive tests (panel A). Loadings >0.60 are in bold type.

| Panel A Component | | | | |
| --- | --- | --- | --- | --- |
|  | Verbal | Processing Speed | Visuospatial |  |
| Vocabulary | **0.840** | 0.366 | 0.237 |  |
| Similarities | **0.820** | 0.191 | 0.406 |  |
| Information | **0.899** | 0.174 | 0.155 |  |
| Block Design | 0.190 | 0.515 | **0.691** |  |
| Matrix Reasoning | 0.423 | 0.333 | **0.616** |  |
| Picture Completion | 0.245 | 0.208 | **0.842** |  |
| Symbol Digit | 0.282 | **0.863** | 0.231 |  |
| Symbol Search | 0.239 | **0.795** | 0.373 |  |

**Panel B.** Rotated component matrix for eight WMS-III memory tests (panel B). Loadings >0.60 are in bold type.

| Panel B Component | | |
| --- | --- | --- |
|  | Episodic memory | Working Memory |
| Logical Memory | **0.750** | 0.318 |
| Verbal Paired Associates | 0.445 | 0.509 |
| Visual Reproduction | **0.730** | 0.208 |
| Faces | **0.699** | 0.238 |
| Family Pictures | **0.809** | -0.019 |
| Letter-Number-Sequencing | 0.343 | **0.681** |
| Digit Span | 0.033 | **0.842** |
| Spatial Span | 0.150 | **0.767** |
